# Behavioral Assessment of Equine Relaxation Following Manual Therapy: A Pilot Study

**DOI:** 10.3390/vetsci12090865

**Published:** 2025-09-05

**Authors:** Yavuzkan Paksoy, Kerem Ural, Hasan Erdoğan, Songül Erdoğan, Serdar Paşa

**Affiliations:** 1 Department of Animal Nutrition and Animal Husbandry, Ceyhan Faculty of Veterinary Medicine, Cukurova University, Adana 01250, Türkiye; 2 Department of Internal Medicine, Faculty of Veterinary Medicine, Aydın Adnan Menderes University, Aydın 09020, Türkiye; kural@adu.edu.tr (K.U.); hasan.erdogan@adu.edu.tr (H.E.); songultp.09@gmail.com (S.E.); pasaserdar@yahoo.co.uk (S.P.)

**Keywords:** manual therapy, behavior, horse health, physiotherapy, stress

## Abstract

The aim of this pilot study was to evaluate the relaxation, stress reduction and behavioral changes observed after manual therapy applied to horses exposed to racing and physical training stimulus. This descriptive approach is aimed at veterinary clinicians to evaluate the therapy process more effectively with behavioral feedback. For this purpose, the study was conducted in two different equestrian clubs in Adana (Adana Mediterranean and Suvari Equestrian Clubs) between 2023 and 2024. A total of 32 racehorses (16 Thoroughbred, 16 Arabian; 16 female, 16 male) of different ages, genders and breeds were included in the study. Five minutes of manual therapy was applied for each of 7 different muscle groups. After the massage, behavioral observations were made for 10 min by moving 2 m away from the animals, and no separate baseline assessment was performed prior to the intervention. The application was carried out by a veterinarian with 15 years of experience. Importantly, no separate baseline assessment or control group was performed, and only behavioral responses were evaluated, which represents a major limitation of this pilot study. Among the observed behaviors in all horses, blinking, muscle twitching, respiratory changes, lip relaxation, licking and chewing were recorded for all horses. Relaxation signs such as head dropping (78.1%), yawning (34.4%), and ears falling to the side (62.5%) were frequently observed. Behaviors such as the appearance of the third eyelid (3.1%), grunting (12.5%) and sneezing (15.6%) were observed at a low percentage. Individual variables such as gender and breed did not have a statistically significant effect on the percentage of behavior (Chi-square test, *p* > 0.05). In conclusion, these preliminary findings suggest that manual therapy applications might be effective in reducing stress by triggering relaxation behaviors in riding horses, as these behaviors have been previously reported in the literature as reliable indicators of relaxation. Evaluation of behavioral responses after massage could be an important tool in determining physiotherapeutic effects. The fact that the application is performed by experienced people is an important factor that increases the success of the therapy and shows that manual therapy provides relaxation regardless of individual differences. Future controlled studies integrating physiological stress biomarkers are warranted to confirm these observations.

## Simple Summary

This study was conducted to evaluate the effects of manual therapy on relaxation and behavioral responses in horses under stress from competition and training. The study was conducted on a total of 32 Thoroughbred and Arabian horses at two different equestrian clubs in Adana. Each animal received an average of 35 min of manual therapy on seven different muscle groups by an experienced veterinarian, followed by 10 min of observation. During the observations, responses such as eye blinking, muscle twitching, lip relaxation, licking, and chewing were observed in all horses. Furthermore, relaxation behaviors such as head dropping (78.1%), yawning (34.4%), and ear drooping (62.5%) were frequently recorded. Individual variables such as sex and breed did not have a significant effect on the incidence of these behaviors. These findings indicate that manual therapy reduces stress and promotes relaxation in horses. These results suggest that behavioral observations can be a practical tool for veterinarians in assessing the effectiveness of physiotherapy interventions. It was concluded that manual therapy can be effective regardless of individual differences when performed by experienced practitioners.

## 1. Introduction

In veterinary medicine, physiotherapy is increasingly used to assist existing treatment protocols, as in human medicine [1]. Active and passive joint movements and mobilization and manipulation practices are among the basic approaches in this field [2]. In particular, manipulation has been shown to provide greater joint range of motion and strength compared to mobilization [3].

Since each horse’s body type, tension level and problems vary, individual evaluation is of great importance in physiotherapy applications. The horse’s behavioral responses after massage and manual therapy provide important clues about the effectiveness of the therapy; these changes become more apparent with observations made after massage [4]. Manual therapy is suggested to influence physiological parameters such as local blood circulation, muscle tone, and autonomic balance, which might contribute to relaxation. When the anatomical connections between body parts are taken into account, for example, the effect of he effect of tension in the head region being reflected in the forelimbs, or conversely, the reflection of a problem originating in the forelimbs being manifested in the head region can be explained through muscle structures [4,5]. Physiotherapy in veterinary medicine is successfully used in many areas such as orthopedic diseases, neurological disorders, musculoskeletal problems, sports injuries, geriatrics and developmental problems [6]. The fact that a horse that was restless or reluctant before the application becomes relaxed and more compliant after the therapy demonstrates the success of physiotherapy [1]. In addition, manual therapy allows the understanding of many problems that cannot be diagnosed with simple methods and contributes to clinical evaluations [7].

Manual therapy is a technique used in musculoskeletal disorders, aimed at improving the functions of muscle, tendon, joint and nerve structures, and is applied by experts [4]. This therapy, which is carried out with techniques such as manipulation and mobilization, plays an active role in muscle relaxation, increasing joint mobility and pain control. Manual therapy is a broad umbrella term that includes manipulation, mobilization, massage, and related techniques. However, the application must be planned specifically for the animal and performed by experts. Otherwise, there may be a risk of damage to the nerve and vascular structures [8]. Different techniques and approaches are used in manual therapy. Methods such as manipulation, mobilization, myofascial release, soft tissue mobilization and traction can be classified under the titles of osteopathy, chiropractic, massage therapy, manual lymph drainage and physiotherapy [7,9]. The main purpose of these methods is to reduce pain, increase mobility, accelerate the healing process and improve the animal’s quality of life.

In a study conducted by Haussler [7], it was emphasized that more research is needed to better understand the local and systemic effects of mobilization and manipulation techniques on healing and relaxation. In this context, this study aimed to determine the relaxation, stress reduction and behavioral changes observed in horses after manual therapy applications. Therefore, this study aimed to evaluate the short-term behavioral effects of massage in horses under controlled conditions.

## 2. Materials and Methods

In this study, routine breeding practices performed in stud farms were not exceeded. Since the data obtained in the study were taken within the scope of these practices, they are not subject to the permission of the Ministry as stated by Republic of Turkey, Adana Governorship, Provincial Directorate of Agriculture and Forestry, Number: E-74530962-325.99-17256991 since they are out of the scope of the Regulation on the Welfare and Protection of Animals Used for Experimental and Other Scientific Purposes published in the Official Gazette dated 13.12.2011 and numbered 28141, according to the provision of the second paragraph of Article 2, “This Regulation does not cover non-experimental agricultural and clinical veterinary practices”. In this study, only a single post-session behavioral assessment was conducted. No control group was created, and no pre-treatment measurements were taken. This preference stems from the descriptive, pilot nature of the study.

In 2023 and 2024, a random selection of horses was used for the research. The study’s animal sample included horses of different ages and gender from various equestrian sports in Adana province (Adana Akdeniz Equestrian Club, Adana Süvari Equestrian Club). 1 January 2023 and 10 August 2024 were the dates of the study. The animals of the study consisted of 32 different horses (16 Thoroughbred, 16 Arabian), each characterized by their unique set of attributes. All horses were actively in race training and performed their routine daily exercise sessions as part of their normal workload. The oldest of these horses was eighteen years old, while the youngest was three. To avoid skewing the statistical analysis of gender-related behavioral tendencies in the results, the study included 16 male and 16 female horses. Horses were housed in similar conditions of care. The horses in shelters stay individually in their stables which are standardly 4 m × 3 m in size. Feeding is carried out in the morning, noon and evening, and their daily feed consisted of 4 kg of oats; 2 kg of barley; 1 kg of ready-made feed with a high vitamin, mineral and protein ratio; 6 carrots; 6 apples; and 10 kg of hay. The total daily feed was divided into three equal portions and provided at 08:00, 14:00, and 20:00. In the farms, water troughs are in the paddocks and stables.

### 2.1. Physical Therapy Process

Olive oil was applied to the massaged areas with 2 hands to soften the areas to be massaged and increase blood circulation. 10 mL of olive oil was used for each muscle section. 70 mL of olive oil was used for each horse. Each horse was massaged for the same muscle groups for an equal amount of time. A total of 7 muscle groups were selected and each muscle group was massaged for 5 min. The massage procedure lasted 5 min for each muscle group, resulting in a total of approximately 35 min per horse, during which all seven major muscle groups were sequentially massaged. Effleurage, petrissage, and friction strokes were performed along the direction of muscle fibers, whereas percussion and vibration were applied more perpendicularly to provide sensory stimulation. After the massage of each muscle group, the horse was moved 2 m away from the vet to allow the horse to relax, feel free and exhibit relaxation behavior. Because it is known that some horses hide the behaviors they want to do when in contact with people. After all muscle areas were massaged, the horse was moved 2 m away from the vet again and watched for 10 min and its behaviors were recorded. The horses were massaged in their own individual stables. In their stables, they were tied to the stable iron with a 1 m long walking rope and kept still. The horse was not attached to an assistant so that it would not hide its behaviors during the massage. The evaluating veterinarian was aware of the treatment allocation. Each horse served as its own control, as measurements were obtained before and after the massage session. A separate negative control group was not included. The massage was performed by Veterinarian Yavuzkan Paksoy, while other researchers waited outside the stable and recorded the horse’s behaviors. The application was performed by Yavuzkan Paksoy, as he had worked at the Turkish Jockey Club Adana Yeşiloba Hippodrome Horse Hospital for 15 years and had a certificate in massage.

**Figure 1:**
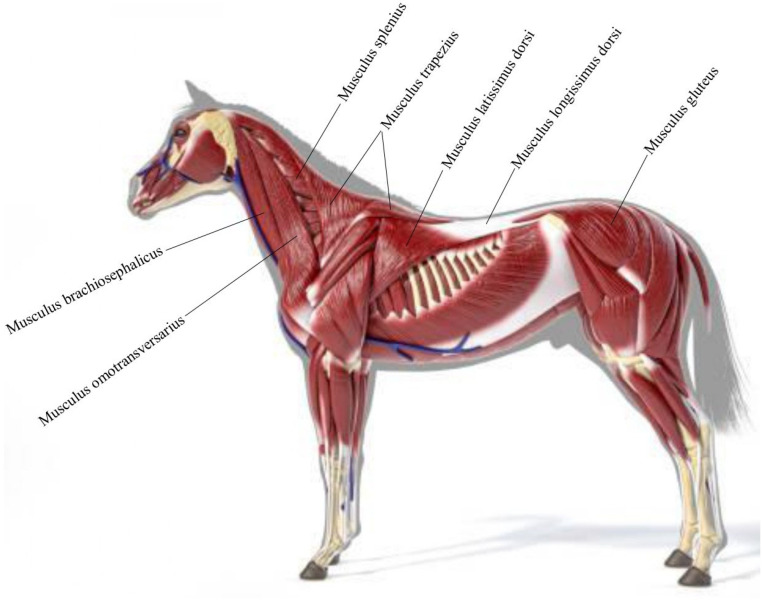
Muscle groups to which massage is applied.

Manual therapy was applied to the horse’s head, neck, back and hindlimb muscle groups. Massage and mobilization techniques were used especially for the muscles connecting the head and forelimb such as *M. brachiocephalicus*, *M. omotransversarius*, *M. splenius*, *M. trapezius*, *M. latissimus dorsi*, *M. longissimus dorsi* located in the back and neck region and *M. gluteus* in the hindlimb (Figure 1). Applications were planned to provide both local relaxation and to evaluate the behavioral effects of the therapy.

The massage application was formed by systematic touch techniques applied to soft tissue. During the massage, the practitioner prepared the body for passive joint stretching by following a calm, careful and regular sequence, aiming to reduce muscle tension. During the application; the effleurage (initial application of oil to the skin without pressure and preparation of the nervous system), petrissage (kneading the muscles), friction (circular pressure movements), percussion (tapping), vibration (vibration applications), and rollen (rolling) techniques were used, respectively. These stages were determined according to previous studies in a way that would maximize the effects of massage on the muscle, fascial system, nervous and circulatory systems [1,5]. After the massage application, behavioral changes exhibited by the animals were observed in order to evaluate the effectiveness of the therapy. The observed behaviors included blinking (rapid closure and reopening of eyelids), body twitching (short local muscle contractions), loosening of the lips (drooping of the lower lip), changes in breathing (visible alterations in respiratory rhythm), sighing (audible or visible deep exhalation), head dropping (lowering of the head below wither level for ≥2 s), general relaxation/softening of facial expression (loss of facial tension and softened eye appearance), licking and chewing (tongue or jaw movements without feed), blowing the nose (forceful exhalation through nostrils), sneezing (sudden nasal expulsion of air), groaning (low vocalization), rocking (gentle shifting of weight from side to side), yawning (wide mouth opening with tongue extension), appearance of the third eyelid (nictitating membrane briefly covering part of the eye), stretching (extension of limbs or body with elongation), fidgeting (small repetitive movements such as pawing), resting on one foot (weight carried by three legs with one fore- or hindlimb flexed), and ears falling to the side (ears positioned laterally >45° from vertical). Each behavior was recorded as present if it occurred at least once during the 10 min observation period, and absent if not observed.

### 2.2. Statistical Analyses

Statistical analyses of the data were performed using the SPSS 29.0 (IBM, New York, NY, USA) program. Descriptive statistics were calculated for each of the behavioral parameters; in this context, the frequencies and percentage distributions of the observed behaviors were determined. Pearson Chi-Square test was used to evaluate the possible relationship between the behaviors and gender and breed variables. Fisher’s Exact Test was applied when more than 20% of the expected cell frequencies were less than 5. The significance level was accepted as *p* < 0.05.

## 3. Results

**Figure 2:**
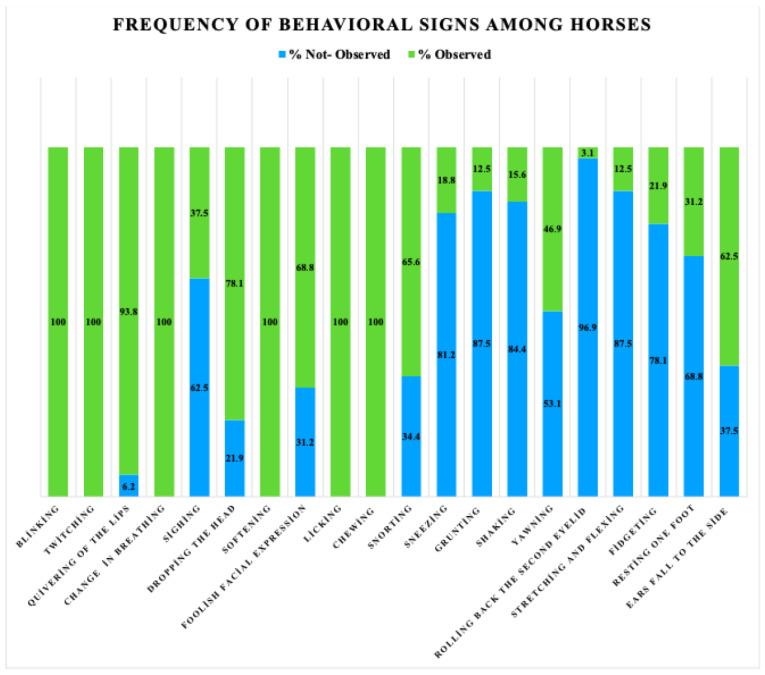
Percentage of behavioral signs among horses.

Among the evaluated behaviors, blinking, twitching, change in breathing, softening, licking and chewing were observed in all individuals at a rate of 100%. In contrast, behaviors such as rolling back of the third eyelid (3.1%), grunting (12.5%), sneezing (15.6%) and shaking (12.5%) were observed at a low percentage. Behaviors with a medium percentage of observation included sighing (37.5%), dropping the head (78.1%) and relaxed facial expression (68.8%). It was also determined that behaviors that might be associated with relaxation or fatigue, such as resting one foot (31.2%) and ears fall to the side (62.5%), were observed at a considerable frequency (Figure 2).

In this pilot study, the percentage of observation of the behavioral parameters evaluated in the study did not show any statistically significant difference in terms of individual characteristics such as gender and breed (*p* > 0.05). No significant relationship was found in the chi-square tests performed in terms of the presence of behavioral symptoms between both gender (male/female) and breed (Thoroughbred/other) groups.

## 4. Discussion

Environmental stress factors that negatively affect the welfare of horses include transportation, exposure to foreign environments, training processes and housing conditions that do not match biological requirements [10]. Although it is often not possible to eliminate such stress factors in racehorses, the application of methods to alleviate their effects is of great importance. In the literature, relaxing massage and music applications have been reported to be beneficial in this context [11,12,13]. All of the animals used in our study were riding horses and they work actively in these situations that require heavy effort and stress and experience the effects of stress intensely.

The negative effects of long-term stress on animal health are well known. Increased emotional reactivity to mild stimuli is observed in horses raised in stressful environments [14,15]. Therefore, determining and controlling stress-related behavioral changes is important for protecting horse welfare. For this purpose, different relaxation techniques, training variety and applications such as acupuncture have been evaluated in recent years [16,17,18,19,20]. In our study, similar to the information above, it was aimed to evaluate the behavioral changes observed after massage in horses exposed to racing and training stress.

In measuring physiological stress responses, parameters such as heart rate (HR) and heart rate variability (HRV) are commonly used [21]. While HR reflects the sympathetic system activity, the root mean square difference (RMSSD) between consecutive heart beats indicates the activity of the parasympathetic (vagal) system [22,23,24]. In addition, measurement of salivary cortisol concentration is a useful method for noninvasive assessment of stress levels [19,25]. In our study, only behavioral changes were evaluated after massage. The fact that measurements such as heart rate and cortisol concentration could not be performed is among the limitations of the study. Another important limitation is the lack of a control group or pre-intervention baseline observations, which prevents attributing the observed changes exclusively to manual therapy. It is also possible that some relaxation-related behaviors might occur when horses are simply tied and left alone, independent of intervention. In addition, the relatively small sample size and the single-session design limit the generalizability of the results. For these reasons, the present findings should be considered preliminary and descriptive, providing a useful basis for future controlled studies that also incorporate physiological markers.

Recent studies investigating the stress-reducing effects of massage therapy in horses have revealed important findings in terms of both physiological and behavioral indicators. Janczarek et al. [16] reported that massage therapy significantly reduced salivary cortisol concentration, decreased heart rate, and decreased the percentage of conflict behaviors, especially tail wagging, ear tilting, and head tossing. In addition, an increase in relaxation behaviors (e.g., half-closed eyes, lip relaxation, chewing, sighing) was observed. In our study, physiological parameters were not measured directly, but the behaviors exhibited by horses after therapy were systematically recorded. The most frequently observed behaviors after massage were eye blinking, licking, chewing, head dropping, yawning, and ear drooping, indicating relaxation.

In previous studies, it was reported that relaxing massages applied three times a week for 25–30 min during the racing season decreased the HR, LF and LF/HF parameters, while increasing the RMSSD and HF values [17,26]. These findings indicate that sympathetic system pressure is reduced and parasympathetic activity is strengthened. In our study, behavioral indicators were used instead of physiological measurements. In addition to behaviors such as licking, chewing, and blinking observed in all individuals after the massage; relaxation signs such as head dropping, yawning and drooping of the ears support the stress-reducing effect of manual therapy, as previously reported as relaxation indicators in horses [11,12,16]. Observing the animals by moving away from before and after the therapy provided more accurate behavioral feedback, and this approach is consistent with the recommendations in the literature [10,17]. Physiotherapy is widely used in veterinary medicine as a complementary method that supports recovery after medical and surgical interventions. The advantages of this method, especially providing effective results in a short time, are among the advantages [27]. It is reported that when applied together with strengthening and balance exercises, it provides a significant increase in the movement capacity of animals [28]. In this context, the relaxation behaviors observed after manual therapy in our study reveal that physiotherapeutic intervention can have a tangible effect in a short time. During massage applications, more stimuli are entered into the brain via the nervous system, and various sensory behaviors occur in the animal accordingly [29,30]. This situation is reflected externally by behaviors such as blinking, dropping the head, licking, chewing and yawning, which are especially associated with relaxation. The fact that these behaviors were observed in almost all individuals in our study shows that massage has an effect that strengthens sensory feedback.

The experience of the practitioner plays an important role in the effectiveness of manual therapy. It has been stated in the literature that practices performed by more experienced physicians reduce pain more and reveal relaxation behaviors more clearly [7]. In this respect, the fact that the practice in our study was performed by a veterinarian who worked in a hippodrome hospital for many years increases the reliability of the behavioral feedback obtained. Approaches such as stroking (effleurage), kneading (petrissage), circular pressure movements (friction), percussion, vibration and rolling (rollen), which are among the manual therapy techniques, are widely used in both human and animal physiotherapy [7]. All of these techniques were applied in a systematic order in our study. In addition, there is a clear difference between the concepts of mobilization and manipulation. While mobilization is the stimulation of soft tissues and joints within the physiological movement limits; manipulation means exceeding these limits in a controlled manner [31]. The massage applied in our study was evaluated within the scope of mobilization techniques.

Behavioral observations made by moving away from the animals after the therapy are consistent with the clinical evaluation methods recommended in the literature. The horse’s behavior provides indirect information about pain, restlessness or relaxation and is considered an important parameter in understanding the therapeutic effect [7]. For this reason, in our study, physical contact with the horse was cut off after the application and the behaviors were allowed to be displayed freely, and all behaviors observed during this time were recorded.

The age, breed, gender and previous medical history of the horse can directly affect the content and duration of the physiotherapy program to be applied [32]. However, since the aim of our study was not to diagnose or treat, but to evaluate the percentage of behavioral responses after therapy, the same techniques and equal durations were applied to all individuals, and the effects of the variables were statistically analyzed. The findings revealed that individual differences such as age, gender or breed did not have a significant effect on the percentage of behavioral responses. This finding shows that manual therapy can create a relaxation effect independent of various individual characteristics [10].

Manual therapy programs can create different responses depending on the physiological and psychological characteristics, genetic structure and relaxation capacity of the individual [9]. Therefore, in our study, the diversity of the sample was increased and individuals of different ages, genders and breeds were included equally. This approach facilitated the generalizability of the results and the evaluation of therapeutic effectiveness [33,34,35].

## 5. Conclusions

Finally, the study findings show that horses exhibit a number of relaxation behaviors during manual therapy applications. In addition, relaxation behavior changes are observed differently in massage sessions applied to painful areas. If these behavioral changes are well known, therapists can diagnose and treat problematic areas in a shorter time. Future studies that incorporate both physiological parameters and appropriate control conditions will be essential to validate and extend these observations.

## Data Availability

The data supporting this study’s findings are available from the corresponding author upon reasonable request.

## References

[1] Prydie D., Hewitt I. (2015). Practical Physsiotherapy for Small Animal Practice.

[2] Cleland J.A., Glynn P., Whitman J.M., Eberhart S.L., MacDonald C., Childs J.D. (2007). Short-term effects of thrust versus nonthrust mobilization/manipulation directed at the thoracic spine in patients with neck pain: A randomized clinical trial. Phys. Ther..

[3] Haussler K.K., Hill A.E., Puttlitz C.M., McIlwraith C.W. (2007). Effect of vertebral mobilization and manipulation on kinematics of the thoracolumbar region. Am. J. Vet. Res..

[4] Masterson J., Reinhold S. (2022). Beyond Horse Massage. A Breakthrought Interactive Method for Alleviating Soreness, Strain and Tension by USET Endurance Team Equine Massage Therapist. The Masterson Method.

[5] Goff L., McGowan C., Stubbs N. (2007). Animal Physiotherapy: Assessment, Treatment and Rehabilitation of Animals.

[6] Altınkaya N., Çağatay S. (2020). Physiotherapy interventions after spinal injuries in dogs: Case reports. İzmir Democr. Univ. Health Sci. J. Iduhes.

[7] Haussler K.K. (2009). Review and manuel therapy techniques in equine practice. J. Equine Vet. Sci..

[8] Memorial Medical Editorial Board. https://memorial.com.tr/saglik-rehberi/manuel-terapi.

[9] Acibadem Web Editorial Board. https://acibadem.com.tr/ilgi-alani/manuel-terapi-nedir/.

[10] Evans D.L. (2007). Welfare of the racehorses during exercise training and racing. The Welfare of Horses.

[11] Kowalik S., Janczarek I., Kędzierski W., Stachurska A., Wilk I. (2017). The effect of relaxing massage on heart rate and heart rate variability in Purebred Arabian racehorses. Anim. Sci. J..

[12] McBride S.D., Hemmings A., Robinson K. (2004). A preliminary study on the effect of massage to reduce stress in the horse. J. Equine Vet. Sci..

[13] Stachurska A., Janczarek I., Wilk I., Kędzierski W. (2015). Does music influence emotional state in race horses?. J. Equine Vet. Sci..

[14] Janczarek I., Kędzierski W. (2011). Emotional response of young race horses to a transfer from a familiar to an unfamiliar environment. Anim. Sci. Pap. Rep..

[15] Janczarek I., Kędzierski W. (2011). Emotional response to novelty and to expectation of novelty in young race horses. J. Equine Vet. Sci..

[16] Janczarek I., Wilk I., Kędzierski W., Stachurska A., Kowalik S. (2016). Off track training ameliorates emotional excitability in Purebred Arabian racehorses. Can. J. Anim. Sci..

[17] Janczarek I., Kędzierski W., Stachurska A., Wilk I. (2016). Can releasing racehorses to paddocks be beneficial? Heart rate analysis—Preliminary study. Ann. Anim. Sci..

[18] Kędzierski W., Janczarek I., Stachurska A. (2012). Emotional response of naive Purebred Arabian colts and fillies to sympathetic and traditional training methods. J. Equine Vet. Sci..

[19] Rizzo M., Arfuso F., Gianetto C., Giudice E., Longo F., Bruschetta D., Piccione G. (2016). Acupuncture needle stimulation on some physiological parameters after road transport and physical exercise. J. Equine Vet. Sci..

[20] Zuo Z.H., Zhang T.Y., Chu J., Zhang Q., Guo Y.X., Shen Z.Q., He C. (2016). Traditional Chinese Medicine in the Treatment of Reproductive Disorders of Large Animals in Asia. Pak. Vet. J..

[21] Riveros-Pinilla D.A., Acevedo G.L., Londoño A.F., Góngora O.A. (2015). Antibodies against spotted fever group Rickettsia sp., in horses of the colombian Orinoquia. Rev. MVZ Córdoba.

[22] Rietmann T.R., Stuart A.E.A., Bernasconi P., Stauffacher M., Auer J.A., Weishaupt M.A. (2004). Assessment of mental stress in warmblood horses: Heart rate variability in comparison to heart rate and selected behavioral parameters. Appl. Anim. Behav. Sci..

[23] Schmidt A., Aurich J., Möstl E., Müller J., Aurich C. (2010). Changes in cortisol release and heart rate variability during the initial training of 3-year-old sport horses. Horm. Behav..

[24] Von Borell E., Langbein J., Desprès G., Hansen S., Leterrier C., Marchant J., Marchant-Forde R., Minero M., Mohr E., Prunier A. (2007). Heart rate variability as a measure of autonomic regulation of cardiac activity for assessing stress and welfare in farm animals—A review. Physiol. Behav..

[25] Loftus L., Marks K., Jones-McVey R., Gonzales J.L., Fowler V.L. (2016). Monty Roberts’ public demonstrations: Preliminary report on the heart rate and heart rate variability of horses undergoing training during live audience events. Animals.

[26] Peeters M., Sulon J., Beckers J.F., Ledoux D., Vandenheede M. (2011). Comparison between blood serum and salivary cortisol concentrations in horses using an adrenocorticotropic hormone challenge. Equine Vet. J..

[27] Wilk I., Kędzierski W., Stachurska A., Janczarek I. (2015). Are results of Crib Opening Test connected with efficacy of training horses in a round-pen?. Appl. Anim. Behav. Sci..

[28] Henderson A.L., Latimer C., Millis D.L. (2015). Rehabilitation and physical therapy for selected orthopedic conditions in veterinary patients. Vet. Clin. Small Anim. Pract..

[29] Risio L. Peripheral nerve injury. Proceedings of the 30th World Small Animal Veterinary Association (WSAVA) Annual Congress 2005.

[30] Samoy Y., Van Ryssen B., Saunders J. (2016). Physiotherapy in small animal medicine. Vlaams Diergeneeskd..

[31] Scaringe J., Kawaoka C., Haldeman S. (2005). Mobilization techniques. Principles and Practice of Chiropractic.

[32] Cauvin E. (1997). Assessment of back pain in horses. Practice.

[33] Ahern T.J. (1994). Cervical vertebral mobilization under anesthetic (CVMUA): A physical therapy for the treatment of cervico-spinal pain and stiffness. J. Equine Vet. Sci..

[34] Brooks J., Colles C., Pusey A., Rossdale P., Green R. (2001). The role of osteopathy in the treatment of the horse. Guardians of the Horse 2.

[35] Sohlberg L., Bergh A., Sternberg-Lewerin S. (2021). A Questionnaire Study on the Use of Complementary and Alternative Veterinary Medicine for Dogs in Sweden. Vet. Sci..

